# Identification and Expression Analysis of GRAS Transcription Factors to Elucidate Candidate Genes Related to Stolons, Fruit Ripening and Abiotic Stresses in Woodland Strawberry (*Fragaria vesca*)

**DOI:** 10.3390/ijms20184593

**Published:** 2019-09-17

**Authors:** Hong Chen, Huihui Li, Xiaoqing Lu, Longzheng Chen, Jing Liu, Han Wu

**Affiliations:** 1 Jiangsu Key Laboratory for the Research and Utilization of Plant Resources, Institute of Botany, Jiangsu Province and Chinese Academy of Sciences, Nanjing 210014, China; 2 Fuyang Academy of Agricultural Sciences, Fuyang 236065, China; 3 Jiangsu Key Laboratory for Horticultural Crop Genetic Improvement, Institute of Vegetable Crops, Jiangsu Academy of Agricultural Sciences, Nanjing 210014, China; 4 State Key Laboratory of Crop Genetics and Germplasm Enhancement, College of Horticulture, Nanjing Agricultural University, Nanjing 210095, China

**Keywords:** genome-wide identification, expression analysis, GRAS transcription factors, stolons, non-climacteric fruit ripening, gibberellins, abiotic stresses, woodland strawberry

## Abstract

The cultivated strawberry (*Fragaria × ananassa*), an allo-octoploid with non-climacteric fleshy fruits, is a popular Rosaceae horticultural crop worldwide that is mainly propagated via stolons during cultivation. Woodland strawberry (*Fragaria vesca*), one of the four diploid progenitor species of cultivated strawberry, is widely used as a model plant in the study of Rosaceae fruit trees, non-climacteric fruits and stolons. One GRAS transcription factor has been shown to regulate stolon formation; the other GRAS proteins in woodland strawberry remain unknown. In this study, we identified 54 FveGRAS proteins in woodland strawberry, and divided them into 14 subfamilies. Conserved motif analysis revealed that the motif composition of FveGRAS proteins was conserved within each subfamily, but diverged widely among subfamilies. We found 56 orthologous pairs of GRAS proteins between woodland strawberry and *Arabidopsis thaliana*, 47 orthologous pairs between woodland strawberry and rice and 92 paralogous pairs within woodland strawberry. The expression patterns of *FveGRAS* genes in various organs and tissues, and changes therein under cold, heat and GA_3_ treatments, were characterized using transcriptomic analysis. The results showed that 34 *FveGRAS* genes were expressed with different degrees in at least four organs, including stolons; only a few genes displayed organ-specific expression. The expression levels of 16 genes decreased, while that of four genes increased during fruit ripening; *FveGRAS54* showed the largest increase in expression. Under cold, heat and GA_3_ treatments, around half of the *FveGRAS* genes displayed increased or decreased expression to some extent, suggesting differing functions of these *FveGRAS* genes in the responses to cold, heat and GAs. This study provides insight into the potential functions of *FveGRAS* genes in woodland strawberry. A few *FveGRAS* genes were identified as candidate genes for further study, in terms of their functions in stolon formation, fruit ripening and abiotic stresses.

## 1. Introduction

Cultivated strawberry (*Fragaria × ananassa*) is a popular Rosaceae horticultural crop worldwide, the fleshy fruit of cultivated strawberry is a non-climacteric fruit [1]. Cultivated strawberry mainly propagates via stolons (also called runners) in agricultural production. However, the complexity of the cultivated strawberry genome (about 805 Mb) has made molecular, genetic and functional studies difficult. Cultivated strawberry is derived from the hybridization of two wild octoploid species (*F.virginiana* and *F.chiloensis*), both of which descended from the merger of four diploid progenitor species (*F. vesca, F.iinumae, F.viridis* and *F.nipponica*) [2]. The genome of *F. vesca* (woodland strawberry) has been published; its small genome (240 Mb) and ease of genetic transformation make it useful as a model plant for the study of Rosaceae fruit trees, non-climacteric fruits and stolons [3]. To date, only a few genes have been demonstrated to regulate stolon formation in woodland strawberry, such as *CONSTANS(CO)* [4], *SUPPRESSOR OF OVEREXPRESSION OF CONSTANS1(SOC1)* [5], *Gibberellin 20-oxidase 4(FveGA20ox4)* [6] and *DELLA* [7,8]. *DELLA* is the only functionally analyzed *GRAS* gene in woodland strawberry [7,8]. Other *GRAS* genes in woodland strawberry remain unidentified and uncharacterized.

The GRAS family was initially named for the first three functionally identified members in *Arabidopsis thaliana*, GIBBERELLIN-INSENSITIVE (GAI), REPRESSOR OF GA1-3 (RGA) and SCARECROW (SCR) [9]. Most GRAS proteins contain a conserved GRAS domain at the C-terminus and a variable region at the N-terminus, but a small number have their GRAS domain at the N-terminus, or have two GRAS domains. In addition, some GRAS proteins, including DELLA, contain a conserved GRAS domain in the C-terminal region and other conserved domains in the N-terminal region [10]. Generally, the conserved GRAS domain contains five ordered conserved motifs: LHR I (leucine heptad repeat I), VHIID, LHR II (leucine heptad repeat II), PFYRE, SAW. LRI, VHIID and LRII, individually and in combination as LRI-VHIID-LRII, are putative DNA-binding sites or protein–protein interaction regions of GRAS proteins, while the functions of the PFYRE and SAW motifs remain unclear [10,11,12]. 

Evolutionary analyses have suggested that GRAS proteins could have originated in bacteria and been transferred into land plants by lateral transfer, and then undergone differentiation and expansion in higher plants [10,13]. Using genome-wide analysis, GRAS family proteins have been identified in numerous plants. According to functional analysis of *GRAS* genes and phylogenetic trees constructed for the model plants *Arabidopsis thaliana* and rice, GRAS proteins can be initially divided into eight subfamilies: LlSCL, PAT1, SCL3, DELLA, SCR, SHR, LS and HAM [14]. However, recent studies have divided GRAS proteins into 10–17 subfamilies based on more detailed data, and about 33–184 GRAS proteins have been identified in various plant species [10,15].

GRAS proteins play important roles in regulating a wide range of developmental and signal transduction processes in higher plants, such as the development of root, shoot, leaf, shoot apical meristem, axillary meristem, flower, embryo and seed and the signaling of gibberellins (GAs), light and stresses [9,16,17]. A large number of *GRAS* genes have been identified and functionally analyzed in plants, especially in *Arabidopsis thaliana* and rice. For example, proteins in the DELLA subfamily are well-known GRAS proteins, and some members have been demonstrated to negatively regulate GA signal transduction [18,19,20]. DELLA proteins also participate in the signaling pathways of auxin, brassinosteroids (BRs), cytokinins (CKs), abscisic acid (ABA), jasmonate (JA) strigolactones (SLs) and ethylene. In fact, DELLA proteins act as a major hub in multiple hormone signaling networks, thereby regulating a variety of developmental processes in plants related to these hormones [20,21]. In addition, AtSCR (belonging to the SCR subfamily) and AtSHR (belonging to the SHR subfamily) in *Arabidopsis thaliana* are involved in various root and shoot development stages, as well as in high salinity and osmotic stress [16]; SCL3 subfamily protein AtSCL3 in *Arabidopsis thaliana* plays a positive role in integrating and maintaining the GA pathway by attenuating DELLA repressors in the root [22,23]; HAM subfamily proteins AtSCL6, AtSCL22 and AtSCL27 can regulate root and leaf development and shoot branching in *Arabidopsis thaliana* [24,25,26]; LAS subfamily proteins MOC1 of rice, AtLAS of *Arabidopsis thaliana* and Ls of tomato are involved in axillary meristem formation [27,28,29,30,31]; DLT subfamily protein GS6 in rice negatively regulates grain size [32] and PAT1 subfamily proteins AtPAT1, AtSCL13 and AtSCL21 act as positive regulators in phytochrome signaling pathways [33,34,35]. 

The stolon of strawberries is above the soil that develops from axillary buds in the aboveground crown, axillary buds can also develop to new rosette stems called branch crowns. An axillary bud will develop into a stolon or a branch crown, depending on environmental conditions such as photoperiod and temperature [36,37]. Potato (*Solanum tuberosum*) also has stolons, but potato stolons are below the soil, which develop only from axillary buds in the belowground shoots under darkness and moist atmosphere conditions, and axillary buds in the aerial part of the shoot mainly develop into leafy shoots [38]. Therefore, strawberry stolons are very different from potato stolons. In woodland strawberry, although the *GRAS* gene *DELLA* has been shown to control stolon formation during asexual reproduction [7,8], knowledge of the other *GRAS* genes remains limited. In this study, we aimed to identify all *GRAS* genes in woodland strawberry and analyze their protein motif compositions, vegetative and reproductive organ expression patterns and responses to cold, heat and GA treatments. According to the findings described above, we selected several *FveGRAS* genes for further functional analysis with respect to stolon formation, fruit ripening and abiotic stress responses in woodland strawberry. This research may provide useful information about the functions of *GRAS* genes in woodland strawberry.

## 2. Results

### 2.1. Identification and Phylogenetic Analysis of GRAS Proteins in Woodland Strawberry

To identify all possible GRAS proteins in woodland strawberry, the protein sequence of the conserved GRAS domain (PF03514.13) was used as a query to identify similar proteins in *Arabidopsis thaliana*, rice and woodland strawberry. After removing redundant results, 34, 60 and 54 GRAS proteins were identified in *Arabidopsis thaliana*, rice and woodland strawberry, respectively. The *GRAS* genes of woodland strawberry were distributed among all seven chromosomes (Chr) as follows: Chr1 (6), Chr2 (5), Chr3 (21), Chr4 (2), Chr5 (6), Chr6 (8) and Chr7 (6). Four *GRAS* gene clusters were located on Chr3, Chr5, Chr6 and Chr7. The largest cluster was on Chr3, where 16 *GRAS* genes (*FveGRAS16–FveGRAS31*) were distributed within an interval of 194 kb. The other three gene clusters each contain two *GRAS* genes: *FveGRAS39* and *FveGRAS40* on Chr5, *FveGRAS42* and *FveGRAS43* on Chr6 and *FveGRAS50* and *FveGRAS51* on Chr7 (Table S1). In addition, the open reading frames (ORFs) of *FveGRAS* genes ranged from 1320 to 4904 bp, and the length of their encoded proteins ranged from 375 to 836 amino acids (aa). Interestingly, the GRAS domains of 53 proteins ranged from 302–416 aa in length, while FveGRAS16 had a GRAS domain of only 83 aa. The molecular weights (kDa) and isoelectric points (pI) of these FveGRAS proteins ranged from 41.46 to 94.21 kDa and 4.64 to 9.20, respectively (Table S1).

**Figure 1:**
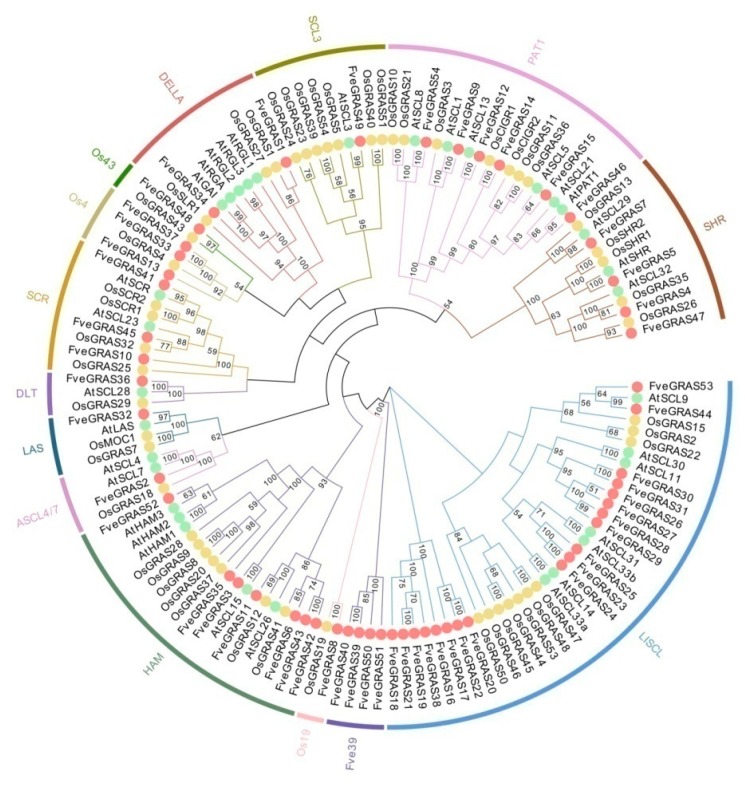
Phylogenetic relationship of GRAS proteins in *Arabidopsis thaliana*, rice and woodland strawberry. 33, 50 and 54 GRAS proteins from *Arabidopsis thaliana*, rice and woodland strawberry were selected to construct phylogenetic tree using MEGA6.0 by the NJ (neighbor-joining) method with the bootstrap test replicated 1000 times. GRAS proteins from *Arabidopsis thaliana*, rice and woodland strawberry are represented by green, yellow and red dots.

We identified 34 and 60 GRAS proteins from *Arabidopsis thaliana* and rice respectively, consistent with previous studies [39,40]. However, one *Arabidopsis thaliana* protein and ten rice proteins were considered to be putative pseudogenes because these members contained partial GRAS domains with missing motifs [39,40]. Therefore, the other 33 and 50 GRAS proteins from *Arabidopsis thaliana* and rice were selected to perform phylogenetic analysis with 54 GRAS proteins from woodland strawberry. On the basis of previous GRAS family studies, we grouped the GRAS proteins of woodland strawberry into 14 subfamilies: SHR, PAT1, SCL3, DELLA, Os43, Os4, SCR, DLT, LAS, ASCL4/7, HAM, Os19, Fve39 and LlSCL (Figure 1). Fifty GRAS proteins identified in woodland strawberry have extensive similarities with proteins from *Arabidopsis thaliana* or rice, and could be divided into 13 subfamilies with high bootstrap values, and the distribution of these GRAS proteins of woodland strawberry among these 13 subfamilies was as follows: SHR (4), PAT1 (6), SCL3 (1), DELLA (2), Os43 (1), Os4 (3), SCR (3), DLT (1), LAS (1), ASCL4/7 (1), HAM (7), Os19 (1) and LlSCL (19). The other four woodland strawberry GRAS proteins (FveGRAS39, 40, 50 and 51) were grouped into a single subfamily, which lacked homologs in either *Arabidopsis thaliana* or rice; we named this new subfamily Fve39. The LlSCL subfamily had the largest number of GRAS proteins in woodland strawberry, and 17 of 19 FveGRAS proteins in the LlSCL subfamily were clustered together into three small branches that lacked homologous proteins in *Arabidopsis thaliana* and rice (Figure 1), suggesting that these genes may be derived from gene duplication events in woodland strawberry.

### 2.2. Conserved Domain and Motif Compositions of GRAS Proteins in Woodland Strawberry

**Figure 2:**
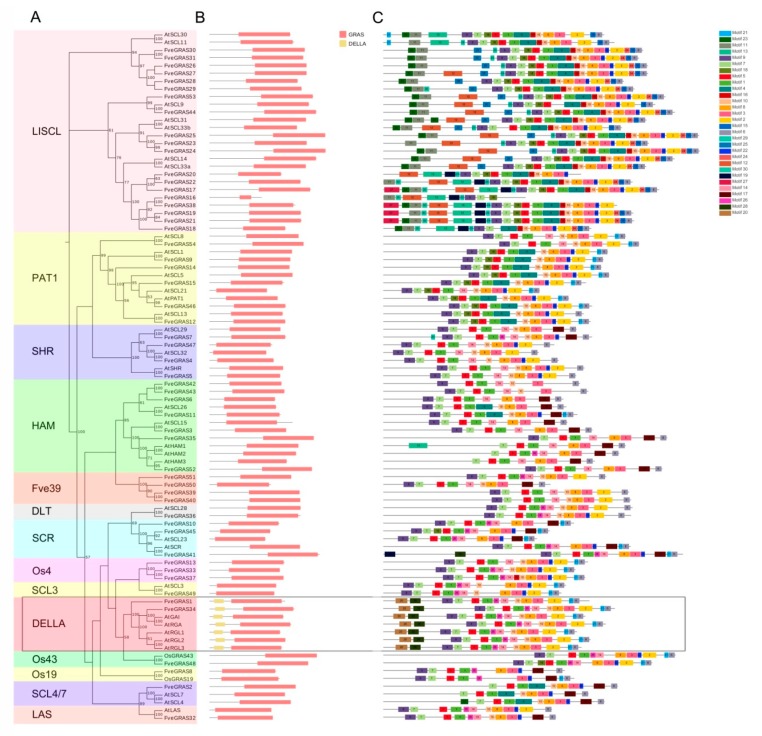
Domain and motif compositions of GRAS proteins from *Arabidopsis thaliana* and woodland strawberry. (**A**) Phylogenetic tree of GRAS proteins in *Arabidopsis thaliana* and woodland strawberry. Different subfamilies are marked with different color backgrounds. (**B**) Domain compositions of GRAS proteins from *Arabidopsis thaliana* and woodland strawberry. Red boxes represent GRAS domains, and yellow boxes represent DELLA domains. Domains were identified by Pfam website. (**C**) Motif compositions of GRAS proteins from *Arabidopsis thaliana* and woodland strawberry. Motifs were identified by MEME software, up to 30 motifs were permitted and other parameters were default settings. Thirty motifs are indicated by different color boxes. The distribution of conserved motifs is presented in Supplementary Table S2. The black rectangle represents DELLA subfamily.

The domain and motif composition of transcription factors are critical to their DNA-binding ability and function. To further elucidate functional conservation and divergence among GRAS subfamilies, we analyzed the conserved domain and motif composition of GRAS proteins in *Arabidopsis thaliana* and woodland strawberry (Figure 2). The results showed that aside from FveGRAS16, all FveGRAS proteins contained a typical GRAS domain (a minimum length of about 350 aa) in the C-terminus, while FveGRAS16 contained only a partial GRAS domain (83 aa), suggesting that it is a pseudogene. In addition, another conserved domain, the DELLA domain, is present in the DELLA subfamily; two FveGRAS proteins (FveGRAS1 and FveGRAS34) and five AtGRAS proteins (AtGAI, AtRGA, AtRGL1, AtRGL2 and AtRGL3) were clustered to this group with a bootstrap value of 100 (Figure 2A,B), suggesting that FveGRAS1 and FveGRAS34 have the same function as DELLA proteins in *Arabidopsis thaliana*.

We used MEME5.0.5 to analyze the motif composition of GRAS proteins in woodland strawberry and *Arabidopsis thaliana*, and found that the C-terminus of GRAS proteins contained many more motifs than the N-terminus, and that GRAS proteins from the same subfamily shared similar motif compositions (Figure 2C); this suggested that GRAS proteins in the same subfamily have similar biological functions. Motifs 1, 3, 5, 6, 7 and 8 were found in all 14 GRAS subfamilies, and are located in the C-terminal region of GRAS proteins, suggesting important roles in the conserved function of the *GRAS* gene family. In addition, motif 2 was completely absent from the HAM, SCR, Os19 and SCL4/7 subfamilies, motif 9 was lost in SCL4/7 and some HAM proteins, motif 10 was missing from the SCR, Os43 and Os19 subfamilies, and some HAM members, and motif 14 was absent from LlSCL, Os19, SCL4/7 and most PAT1 proteins. Meanwhile, motif 14 was found only in LlSCL, PAT1 and SCL4/7, motif 18 was present in most members of LlSCL and PAT1; motifs 11, 15, 16, 23, 24 and 29 were found only in members of LlSCL, and motifs 20 and 28 were only found in DELLA proteins (Figure 2C; Table S2). In conclusion, the diversity in motif number and composition among GRAS subfamilies reveals that the functions of GRAS proteins likely diverged during evolution.

### 2.3. Identification of Orthologous and Paralogous GRAS Genes in Woodland Strawberry, Arabidopsis thaliana and Rice

**Figure 3:**
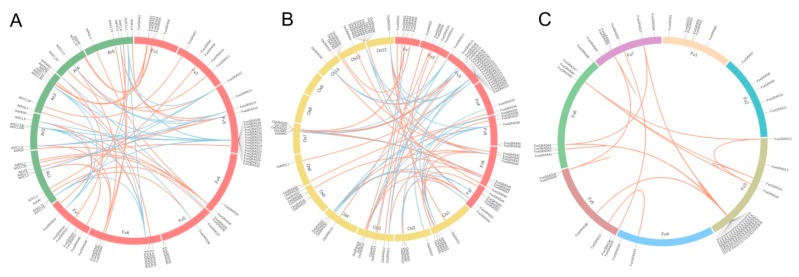
Orthologs, co-orthologs and paralogs of *GRAS* genes from woodland strawberry, *Arabidopsis thaliana* and rice. (**A**) Orthologs and co-orthologs of *GRAS* genes between *Arabidopsis thaliana* and woodland strawberry. (**B**) Orthologs and co-orthologs of *GRAS* genes between rice and woodland strawberry. (**C**) Paralogs of *GRAS* genes in woodland strawberry. OrthoMCL was used to identify orthologs, co-orthologs and paralogs. Red, green and yellow boxes in (**A**) and (**B**) represent chromosomes of woodland strawberry, *Arabidopsis thaliana* and rice. Red and blue lines in (**A**) and (**B**) represent orthologous and co-orthologous gene pairs. Different color boxes in (**C**) represent the seven chromosomes of woodland strawberry, and red lines in (**C**) represent paralogous gene pairs. The distribution of orthologs, co-orthologs and paralogs of *GRAS* genes are presented in Supplementary Tables S3 and S4.

To compare the genetic relationships of *GRAS* genes in woodland strawberry to those of *Arabidopsis thaliana* and rice, we used OrthoMCL to identify orthologous and co-orthologous genes among the three plant genomes, as well as paralogous genes within woodland strawberry (Figure 3). The results showed 56 orthologous and 75 co-orthologous gene pairs of GRAS proteins between woodland strawberry and *Arabidopsis thaliana* (Figure 3A), and 47 orthologous and 118 co-orthologous gene pairs between woodland strawberry and rice (Figure 3B). Furthermore, 23 *FveGRAS* genes had orthologous genes in both *Arabidopsis thaliana* and rice, nine *FveGRAS* genes had orthologous genes in *Arabidopsis thaliana* but not in rice, eight *FveGRAS* genes had orthologous genes in rice but not in *Arabidopsis thaliana* and 14 *FveGRAS* genes did not have orthologous genes in either *Arabidopsis thaliana* or rice (Table S3). Additionally, 92 paralogous gene pairs were identified in the woodland strawberry, of which 77 pairs were in the LlSCL subfamily (Figure 3C; Table S4), suggesting that *LlSCL* family genes in woodland strawberry have undergone gene duplication events during the process of evolution.

### 2.4. Expression Profile Analysis of GRAS Genes in Various Organs of Woodland Strawberry

**Figure 4:**
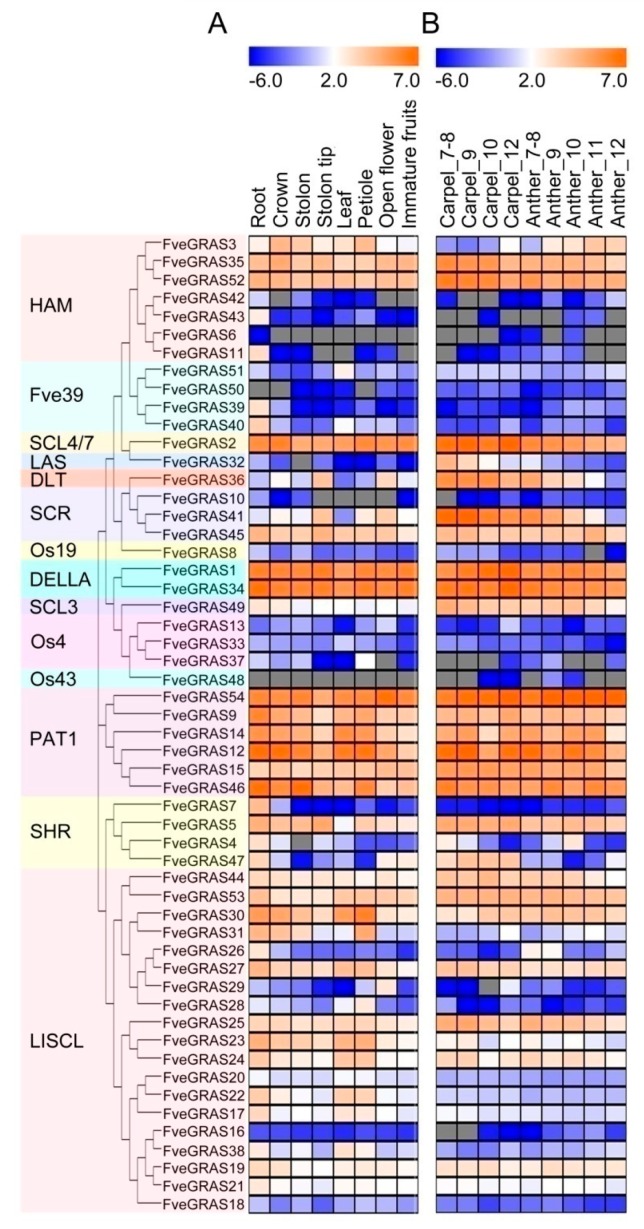
Expression pattern of *GRAS* genes in various organs of woodland strawberry. (**A**) Expression pattern of *GRAS* genes in the root, crown, stolon, stolon tip, leaf, petiole, open flower and immature fruit of woodland strawberry. Samples were collected in our lab, and then RNA-seq was performed. (**B**) Expression pattern of *GRAS* genes in the developing carpel and anther of woodland strawberry. The transcriptome data was downloaded from Li et al. (2019) [43]. Fragments per kilobase per million (FPKM) values in (**A**) and reads per kilobase per million (RPKM) values in (**B**) were in Supplementary Tables S5 and S6, and the heat map showed log2 level. Numbers after carpel and anther represent the developmental stages. The details of the stages were on the website (http://bioinformatics.towson.edu/strawberry/newpage/Tissue_Description.aspx. Access date: 25 May 2019).

To investigate the expression profiles of *FveGRAS* genes in various organs of woodland strawberry, we collected roots, crowns, stolons, stolon tips, leaves, petioles, open flowers and immature fruits of the woodland strawberry variety “Hawaii 4” for transcriptomic analysis. *FveGRAS* genes enumerated in normalized fragments per kilobase per million (FPKM) were selected for display in a heat map of expression profiles. An FPKM value greater than 1 indicates that the gene is expressed [41,42]. Our results showed that six *FveGRAS* genes (*FveGRAS6*, *50*, *32*, *10*, *48* and *16*) have FPKM values lower than 1 in all eight organs, while the other 48 *FveGRAS* genes have FPKM values greater than 1 in at least one organ (Table S5). The detailed results were as follows: 34 *FveGRAS* genes were expressed to varying degrees in at least four organs, including nine *FveGRAS* genes (*FveGRAS35*, *52*, *2*, *1*, *34*, *54*, *12*, *15* and *46*) with FPKM greater than 10 in all eight organs; this suggests that any of these *GRAS* genes may play roles in the growth and development of several organs in woodland strawberry. We found that only a few genes showed organ-specific expression, with seven genes (*FveGRAS42*, *43*, *11*, *39*, *8*, *7* and *26*) having the highest expression in roots and nearly no expression in other seven organs; this indicates that these genes may participate in root growth and development. *FveGRAS13* and *FveGRAS29* were the only genes showing elevated expression solely in open flowers, and *FveGRAS33* had high expression in leaves. *FveGRAS4* had its highest expression level in roots, and moderate expression in crowns and stolon tips. *FveGRAS37* was expressed mainly in petioles, at levels about three-fold greater than in roots and stolons. *FveGRAS51* was most highly expressed in leaves, and showed moderate expression in roots and open flowers (Figure 4A). In conclusion, the expression profiles of *FveGRAS* genes in various organs suggest that numerous *FveGRAS* genes play roles in organ growth and development in woodland strawberry.

The expression levels of *FveGRAS* genes in developing carpels and anthers were investigated using published RNA-sequencing (RNA-Seq) data [43]. The results showed that 32 *FveGRAS* genes had higher expression levels than the other 22 genes (Table S6). Specifically, the expression levels of four genes (*FveGRAS3*, *54*, *47* and *31*) were elevated, while those of 15 genes (*FveGRAS35*, *52*, *32*, *36*, *41*, *45*, *49*, *12*, *15*, *4*, *44*, *25*, *23*, *24* and *17*) were reduced in carpel-12 compared to carpel-7; the same pattern was observed in the anther. Six gene expression levels (*FveGRAS2*, *1*, *34*, *9*, *14* and *30*) were higher in carpel-12 than carpel-7, but decreased in the anther. Two genes (*FveGRAS46* and *27*) showed reduced expression in carpel-12 compared to carpel-7, but increased expression in the anther; three other genes (*FveGRAS5*, *53* and *26*) had similar expression levels in the developing carpel, but slightly decreased expression in the developing anther. Furthermore, some fluctuations in *FveGRAS* gene expression were noted during carpel and anther development. For example, the expression of *FveGRAS2* at carpel-10 was lower than that at carpel-7, but the level at carpel-12 was higher than at carpel-7. This gene expression pattern of “first decrease and then increase” also occurred in *FveGRAS34*, *9*, *14*, *12*, *23* and *17* in the carpel. A greater number of genes displayed a “first increase and then decrease” pattern during the development of carpel and anther, such as *FveGRAS3*, *45*, *1*, *49*, *54*, *9*, *46*, *5*, 4, *30* and *31* in anther and *FveGRAS41* in carpel (Figure 4B). Therefore, different *GRAS* genes play different roles in the development of the woodland strawberry carpel and anther.

### 2.5. Expression Analysis of GRAS Genes in Developing and Ripening Fruits of Woodland Strawberry

**Figure 5:**
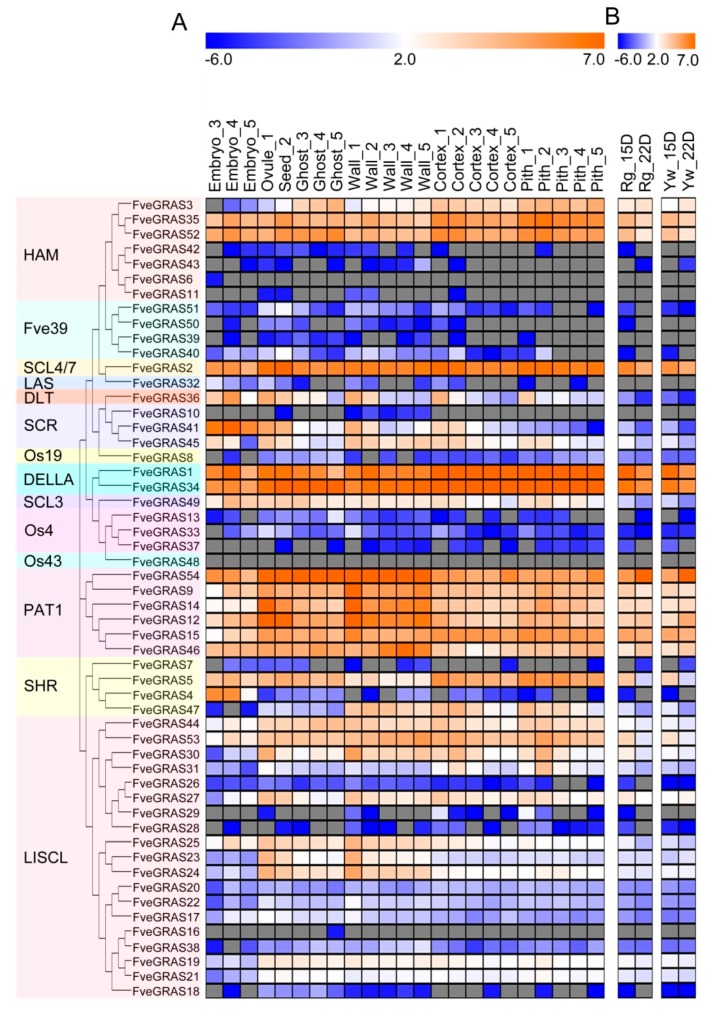
Expression pattern of *GRAS* genes in developing and ripening fruit tissues of woodland strawberry. (**A**) Expression pattern of *GRAS* genes in developing fruit tissues (including embryo, ovule, ghost, wall, cortex and pith). (**B**) Expression change of *GRAS* genes from immature to ripening fruits in two varieties “Ruegen” and “Yellow Wonder”. RPKM values in (**A**,**B**) downloaded from Li et al. (2019) [43] were in Supplementary Tables S7 and S8, and the heat map showed log2 level. Numbers after tissues in (**A**) represent different developmental stages: Stage 1 (pre-fertilization), stage 2 (2–4 days post-anthesis (DPA)), stage 3 (6–9 DPA), stage 4 (8–10 DPA) and stage 5 (10–13 DPA). Rg and Yw in (**B**) represent “Ruegen” and “Yellow Wonder”, and 15D and 22D represent 15 and 22 DPA.

The period from anthesis to green fruit (including achene and receptacle) is divided into five stages: Stage 1 (pre-fertilization), stage 2 (2–4 days post-anthesis (DPA)), stage 3 (6–9 DPA), stage 4 (8–10 DPA) and stage 5 (10–13 DPA). The achene can be divided into the wall and seeds, the latter of which are further divided into embryo and ghost (seeds without an embryo) [44]. The heat map of *FveGRAS* gene expression based on published RNA-Seq data [43] showed that from stage 1 to stage 2, the expression levels of seven genes (F*veGRAS3*, *35*, *52*, *51*, *40*, *34* and *5*) increased markedly, by more than 30%, while the expression of 16 genes (*FveGRAS36*, *41*, *45*, *33*, *9*, *14*, *12*, *15*, *30*, *31*, *27*, *25*, *23*, *24*, *22* and *17*) decreased by more than 20% in seed 2 (vs. ovule 1; Figure 5A; Table S7), suggesting that the expression of these *FveGRAS* genes was affected by fertilization. Then, from stage 3 to stage 5, the expression of 14 *FveGRAS* genes (*FveGRAS35*, *52*, *2*, *36*, *41*, *1*, *34*, *49*, *9*, *5*, *44*, *53*, *25* and *24*) first increased at embryo 4 and then decreased at embryo 5; four genes (*FveGRAS32, 45, 54* and *4*) continuously decreased, and seven genes (*FveGRAS14*, *12*, *15*, *46*, *30*, *27* and *17*) continuously increased in the embryo from stage 3 to stage 5. However, the same genes showed distinct expression patterns in ghost, where in five gene expressions (*FveGRAS3*, *52*, *54*, *44* and *30*) increased, in 15 gene expressions (*FveGRAS36*, *41*, *45*, *1*, *34*, *49*, *14*, *12*, *53*, *31*, *27*, *25*, *23*, *24* and *19*) decreased, in three gene expressions (*FveGRAS35*, *2* and *5*) first decreased then increased, and in two gene expressions (*FveGRAS15* and *46*) first increased and then decreased (from stage 3 to stage 5). Furthermore, in the achene wall from stage 1 to stage 5, the expression levels of four genes (*FveGRAS3*, *34*, *54* and *53*) increased, while those of 13 genes (*FveGRAS36*, *41*, *33*, *15*, *44*, *30*, *31*, *27*, *25*, *23*, *24*, *22* and *17*) decreased; furthermore, eight genes (*FveGRAS35*, *52*, *40*, *1*, *49*, *46*, *5* and *47*) showed the “first increase and then decrease” expression pattern, and five genes (*FveGRAS2*, *45*, *9*, *14* and *12*) showed the “first decrease and then increase” expression pattern. Notably, the greatest increase or decrease in expression for most *FveGRAS* genes occurred from wall 1 to wall 2 (Figure 5A; Table S7), suggesting that fertilization of the ovule also affects *FveGRAS* gene expression levels in the achene wall.

The receptacle of woodland strawberry fruits was divided into cortex and pith [44], and the result of published RNA-Seq data [43] showed that most *FveGRAS* genes had similar expression patterns in the cortex and pith among stages 1 to 5 (Figure 5A; Table S7). In both cortex and pith, 18 genes (*FveGRAS35*, *52*, *2*, *36*, *41*, *45*, *1*, *49*, *12*, *15*, *5*, *47*, *44*, *27*, *29*, *25*, *23* and *24*) showed decreased expression, four genes (*FveGRAS34*, *53*, *30* and *31*) showed the “first increase and then decrease” expression pattern, and two genes (*FveGRAS54* and *46*) showed the “first decrease and then increase” expression pattern. The expression of *FveGRAS3* decreased in the cortex, but increased in the pith, while *FveGRAS9* and *FveGRAS14* expression decreased markedly in the cortex, but first increased and then decreased in pith (Figure 5A; Table S7). These results suggest that these *FveGRAS* genes play roles in the early development of fruits in woodland strawberry.

We also analyzed changes in the expression of *FveGRAS* genes between immature and ripening fruits in two woodland strawberry varieties, “Ruegen” (red fruit) and “Yellow Wonder” (yellow fruit) using published RNA-seq data [43]. The results showed that 26 *FveGRAS* genes had relatively high expression levels, among which 16 genes showed decreased, and only four genes (*FveGRAS3*, *54*, *12* and *46*) showed increased expression in both “Ruegen” and “Yellow Wonder” between immature and ripening fruits (Figure 5B; Table S8). Notably, the expression of *FveGRAS54* increased by more than 10-fold in ripening fruits compared to immature fruits, suggesting that *FveGRAS54* may play an important role in fruit ripening. The expression of another gene, *FveGRAS27*, decreased in “Ruegen”, but showed a slight increase in “Yellow Wonder” (Figure 5B; Table S8), suggesting that this gene may have different roles in the ripening processes of red versus yellow fruits.

### 2.6. Expression Analysis of GRAS Genes of Woodland Strawberry under Cold and Heat Stresses

**Figure 6:**
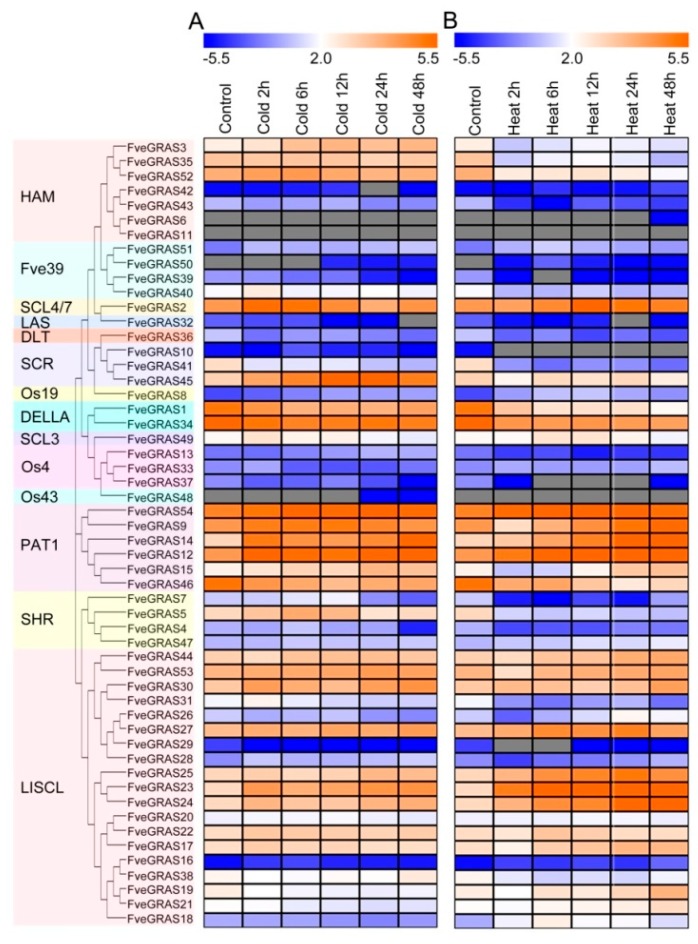
Expression changes of *GRAS* genes of woodland strawberry under cold and heat stresses. (**A**) Expression changes of *GRAS* genes of woodland strawberry under cold stress. (**B**) Expression changes of *GRAS* genes of woodland strawberry under heat stress. Woodland strawberry variety “Hawaii 4” seedlings were used for cold (4 °C) and heat (40 °C) treatments. FPKM values were in Supplementary Table S9, and the heat map showed log2 level.

To assess the functions of *FveGRAS* genes in plant defenses against abiotic stresses, we performed a transcriptomic analysis using “Hawaii 4” seedlings to analyze changes in the expression of *SlGRAS* genes under cold and heat stresses. In cold-treated seedlings, the FPKM values of 36 *FveGRAS* genes were greater than 1. Among these genes, the expression levels of 24 genes (*FveGRAS3, 52, 51, 40, 2, 45, 49, 54, 9, 14, 12, 15, 7, 5, 47, 44, 53, 30, 27, 25, 23, 24, 22* and *17*) were elevated, of which 12 gene expressions (*FveGRAS3, 51, 2, 45, 14, 12, 15, 7, 5, 30, 23* and *24*) increased by more than two-fold. Especially, *FveGRAS45* expression increased by 5.6-fold at 24 h, and *FveGRAS14* expression increased by 5.6-fold at 48 h. In addition, ten genes (*FveGRAS36, 41, 1, 34, 46, 31,26, 38, 19* and *21*) showed reduced expression, of which seven gene expressions (*FveGRAS36, 41, 1, 46,31, 26* and *21*) decreased by more than two-fold (Figure 6A; Table S9). Under heat stress, the FPKM values of 37 *FveGRAS* genes were greater than 1. The expression levels of 18 genes (*FveGRAS51, 2, 49, 54, 14, 12, 47, 44, 30, 27, 25, 23, 24, 22, 17, 19, 21* and *18*) were elevated compared with the control, of which 12 gene expressions (*FveGRAS51, 2, 54, 14, 12, 47, 27, 25, 23, 24, 19* and *18*) increased by more than two-fold. Especially, *FveGRAS14* and *FveGRAS23* expressions increased by about 19- and 16-fold at 48 h, respectively (Figure 6B; Table S9). In addition, the expressions of 18 genes (*FveGRAS3, 35, 52, 40, 36, 41, 45, 1, 34, 9, 15, 46, 7, 5, 53, 31, 26* and *38*) were reduced compared with the control, of which 11 genes (*FveGRAS3, 35, 52, 40, 36, 41, 1, 46, 7, 5* and *31*) were reduced at least five-fold at a certain hour post treatment (Figure 6B; Table S9). 

**Figure 7:**
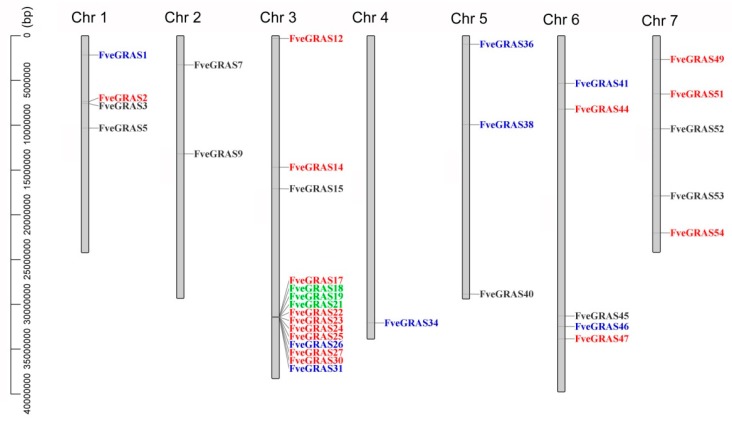
Chromosomal positions of woodland strawberry *GRAS* genes that respond to cold and/or heat treatments. Fifteen up-regulated genes and eight down-regulated genes both in cold and heat treatments are indicated by red and blue fonts, respectively. Nine genes with increased expression in cold stress, but decreased expression in heat stress are indicated by black fonts, and three genes with decreased expression in cold stress, but increased expression in heat stress are indicated by green fonts.

Notably, 15 gene expressions (*FveGRAS51, 2, 49, 54, 14, 12, 47, 44, 30, 27, 25, 23, 24, 22* and *17*) were increased, and eight gene expressions (*FveGRAS36, 41, 1, 34, 46, 31, 26* and *38*) were decreased both in cold and heat treatment seedlings, which distributed among six chromosomes: Chr1 (2), Chr3 (11), Chr4 (1), Chr5 (2), Chr6 (4) and Chr7 (3). In addition, nine gene expressions (*FveGRAS3, 52, 40, 45, 9, 15, 7, 5* and *53*) were increased by cold treatment, but decreased by heat treatment, and three gene expressions (*FveGRAS19, 21* and *18*) were decreased by cold treatment, but increased by heat treatment, these 12 genes distributed among six chromosomes: Chr1 (2), Chr2 (2), Chr3 (4), Chr5 (1), Chr6 (1) and Chr7 (2) (Figure 7). The result suggests that *GRAS* genes have multiple roles in responses to cold and heat stresses.

### 2.7. Analysis of GRAS Gene Expression during Woodland Strawberry Responses to GA Phytohormone.

**Figure 8:**
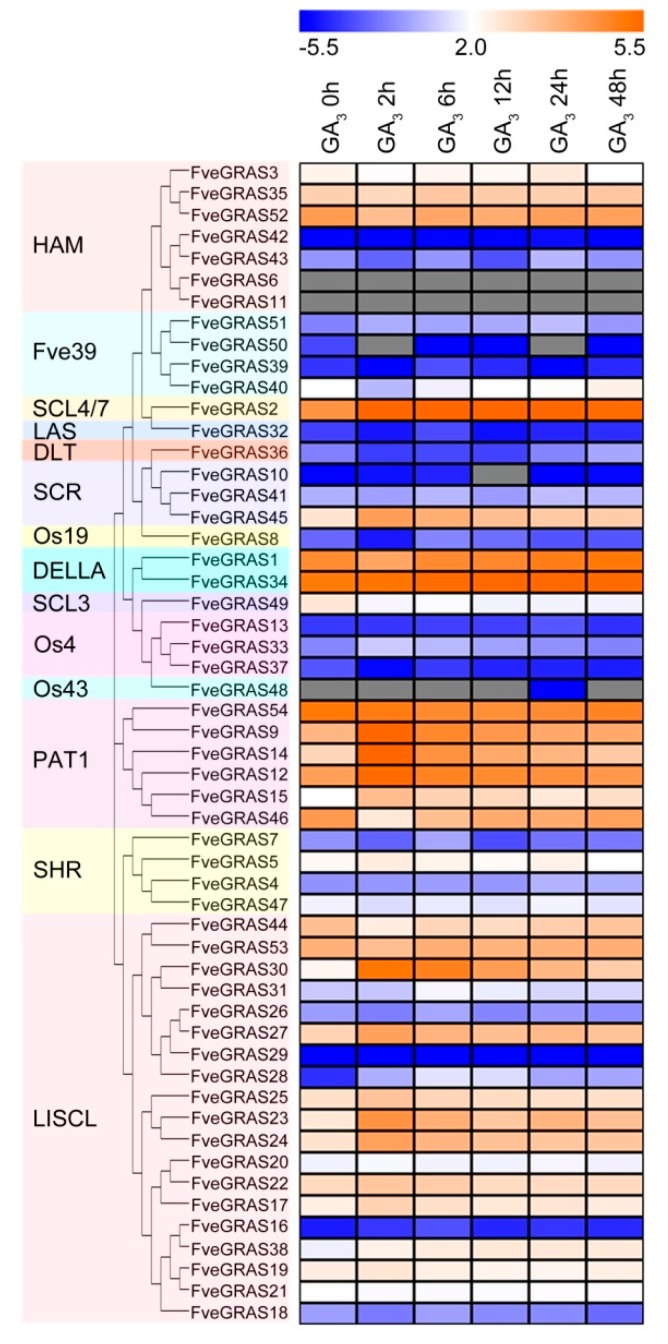
Expression changes of *GRAS* genes of woodland strawberry under GA_3_ treatment. Woodland strawberry variety “Hawaii 4” seedlings were used for GA_3_ treatment. FPKM values were in Supplementary Table S10, and the heat map showed log2 level.

The differentiation and elongation of stolons in strawberry are regulated by the phytohormone group known as GAs, and many *GRAS* genes are involved in the response to GAs. Therefore, we used woodland strawberry seedlings treated with exogenous GA_3_ to analyze the response of *FveGRAS* genes to GAs. The results showed that after GA_3_ treatment, the expression levels of nine genes (*FveGRAS52, 40, 1, 49, 54, 46, 47, 44* and *53*) first decreased, and then increased, including three genes (*FveGRAS40, 46* and *44*) that were reduced by more than two-fold at 2 h, and began to increase thereafter(Figure 8; Table S10). In addition, the expression of 17 genes (*FveGRAS2, 45, 34, 9, 14, 12, 15, 5, 30, 31, 27, 25, 23, 24, 22, 17* and *38*) first increased and then decreased, including ten genes (*FveGRAS2, 45, 9, 14, 12, 15, 30, 27, 23* and *24*) with a more than two-fold increase in expression level at 2 h, which later began to decrease. Especially, *FveGRAS14* and *FveGRAS30* had the highest increase by about 14- and 7-fold at 2 h, respectively (Figure 8; Table S10). This result showed that GA-regulated *FveGRAS* genes might be involved in GA-related biological processes in woodland strawberry.

## 3. Discussion

### 3.1. Evolution and Expansion of GRAS Genes

To date, GRAS proteins have been identified in many higher plants, as well as in lower plants such as the lycophyte *Selaginella moellendorffii* and the bryophyte *Physcomitrella patens* [45,46], as well as in the aquatic alga *Spirogyra pratensis*, which belongs to the charophytes, the ancestral taxon of all land plants [46]. Interestingly, GRAS proteins have also been found in some bacteria, but not in any fungi, Metazoa or other algae, suggesting that horizontal gene transfer (HGT) of the GRAS domain from a bacterial source to the common ancestor of land plants is possible and may explain the origin of plant *GRAS* genes [47]. Only one or two GRAS homologs appear to be present per bacterial genome, but at least 30 *GRAS* genes have been identified in all higher plant species investigated. Moreover, bacterial GRAS proteins clearly cluster into a separate clade, rather than the clades of plants, suggesting that *GRAS* genes have undergone differentiation and expansion in higher plants, which may have aided their adaptation to the land environment [10,13,47]. 

Over the last two decades, several *GRAS* genes have been isolated and functionally analyzed, especially in the model plants *Arabidopsis thaliana* and rice. *GRAS* genes are continuously being found in various plants using genome-wide identification methods. The number of *GRAS* genes in plants of various species ranges widely, 33–184 GRAS proteins have been identified in various plant species, and these genes are clustered into 8–17 subfamilies, depending on the study [10,15]. In woodland strawberry, we identified 54 *GRAS* genes, and divided them into 14 subfamilies based on similar motif composition (Figure 1 and Figure 2); of these subfamilies, four (Os4, Os19, Os43 and Fve39) were absent in *Arabidopsis thaliana*. The GRAS proteins Os4, Os19 and Os43 can be found in other plants, such as rice [14], *Populus* [39], tomato [48], castor beans [49], *Prunus mume* [50] and tea plant [15], and we therefore suggest that these three gene families existed before the divergence of dicotyledons and monocotyledons, but were lost in *Arabidopsis thaliana*. Interestingly, the Fve39 family appears to be a woodland strawberry-specific family (Figure 1), because no orthologous genes were found in *Arabidopsis thaliana* or rice (Figure 3). Some other plant species have similar specific families, such as the Pt20 family in *Populus* and tomato [39,48], G_GRAS in cotton [51], and Rc_GRAS in castor beans [49]; other plant species do not have specific families, including maize [52], tea [15] and Chinese cabbage [53]. Therefore, we speculated that these species-specific families originated from ancestors that occurred after the divergence of dicotyledons and monocotyledons, or arose from ancestors before the divergence of dicotyledons and monocotyledons but were entirely lost in some species during their evolutionary process, such as *Arabidopsis thaliana*, rice and so on. It is tempting to speculate that the genes in the Fve39 subfamily play special roles in woodland strawberry.

Another notable finding is that the LlSCL subfamily contains the largest number of *GRAS* genes. Expansion of the *LlSCL* subfamily genes has been suggested to occur independently in multiple plant species, as *LlSCL* genes within a given plant cluster into an independent group [54], such as those in rice [14], tea [15], tomato [48] and *Populus* [39], as well as in woodland strawberry, as observed in this study. In total, 35% (19 of 54 genes) of the *GRAS* genes in woodland strawberry belong to the LlSCL subfamily, which is a much higher proportion than in other plants, such as *Arabidopsis thaliana* (21%, 7/33) [14], rice (20%, 10/50) [14], cotton (13%, 20/150) [51], tea plant (19%, 10/52) [15], castor beans (15%, 7/46) [49] and *Populus* (11%, 12/106) [39], indicating that a marked expansion of *GRAS* genes may have occurred in the LlSCL subfamily during the evolution of the woodland strawberry. Consistent with this, 16 *GRAS* genes (*FveGRAS16–FveGRAS31*) formed a large gene cluster on chromosome 3 (Table S1), showing that the expansion of *GRAS* genes in the LlSCL subfamily may have been due to tandem gene duplication events. Duplications have long been considered a primary source of genetic redundancy, as well as functional novelty, leading to new gene functions and expression patterns [55]. Accordingly, we found differing *LlSCL* subfamily gene expression patterns between woodland strawberry and *Arabidopsis thaliana*. All *LlSCL* subfamily genes (*AtSCL9, 11, 14, 30, 31* and *33*) in *Arabidopsis thaliana* were expressed in all tested organs (roots, leaves, flowers and seedlings) [56]. In woodland strawberry, 13 genes in the *LlSCL* subfamily (*FveGRAS44, 53, 30, 27, 25, 23, 24, 20, 22, 17, 38, 19* and *21*) that were orthologous with *Arabidopsis thaliana* genes were expressed to varying degrees in all tested organs, while the other six *LlSCL* subfamily genes, which were not orthologous with *Arabidopsis thaliana* genes, showed organ-specific expression or negligible expression (Figure 4A). Therefore, duplication of *GRAS* genes in the *LlSCL* family may have produced new functions related to the growth and development of woodland strawberry compared to orthologous genes in *Arabidopsis thaliana*.

### 3.2. Possible Roles of GRAS Genes in the Vegetative Organs of Woodland Strawberry

Several *GRAS* genes have been functionally characterized, particularly in *Arabidopsis thaliana*, demonstrating that *GRAS* family genes play important roles in a wide variety of biological process. However, few *GRAS* genes have been studied in woodland strawberry, so the expression patterns of *GRAS* genes identified herein could help in the assessment of their possible functions in woodland strawberry. The *AtSHR* and *AtSCR* genes in *Arabidopsis thaliana* have received the most research attention in the *GRAS* family; these genes can interact with each other, or with other proteins, to regulate root, shoot and leaf development, and play similar roles in rice [16]. In addition, other subfamilies of genes in the *GRAS* family have been found to participate in root meristem arrest, lateral root development, root modification and development of the leaf and shoot apical meristem, including the *HAM* subfamily genes *AtSCL6, 22* and *27* (in *Arabidopsis thaliana*), *MtNSP2* (in *Medicago truncatula*), *PhHAM* (in petunia), the SCL3 subfamily gene *AtSCL3* (in *Arabidopsis thaliana*) and the *PAT1* subfamily gene *SIN1* (in *Phaseolus vulgaris*) [16,17]. This study showed that all of these subfamilies contain some genes expressed in the root, stem, leaf and petiole of woodland strawberry (Figure 4A), suggesting conserved functions with homologous genes in other plant species, whereas other genes (*FveGRAS42, 43, 6, 11, 10* and *7*) in these subfamilies were specifically expressed in roots or were undetectable (Figure 4A), showing that these genes may be involved in root development or other biological processes. 

The cultivated strawberry plant propagates sexually through seeds and vegetatively through stolons. Stolon propagation is the main method used in cultivated strawberry production. The model plants *Arabidopsis thaliana*, rice and tomato do not produce stolons, so diploid woodland strawberry is an ideal model for studying the mechanism of stolon formation. CO, SOC1, FveGA20ox4 and DELLA have been demonstrated to regulate stolon formation [4,5,6,7,8]. DELLA proteins belong to the GRAS family, and act as repressors of GA signaling, thereby regulating numerous processes during growth and development [19]. Five FveGRAS proteins (gene06210, gene30958, gene01356, gene22702 and gene06947) were identified as probable DELLA subfamily proteins [7,44], but only gene06210 (FveGRAS34 in this study) and gene30958 (FveGRAS1 in this study) belonged to the DELLA subfamily in our study; gene01356, gene22702 and gene06947 were grouped into the Os43 and Os4 subfamilies. Furthermore, only gene06210 (FveGRAS34) contains a full DELLA motif, and mutation of gene06210 in a runnerless variety (Yellow Wonder) rescues the ability to develop runners [7], in addition, silencing the expression of *FveRGA1* gene (*DELLA*, *gene06210*) in the naturally non-runnering woodland strawberry cultivars “Ruegen” and “Yellow Wonder” produced many runners [8], demonstrating that this DELLA protein controls runner formation during asexual reproduction in woodland strawberry [7,8]. We found that *gene06210* (*FveGRAS34*) was highly expressed in all tested organs (Figure 4A), and is the only gene containing a full DELLA motif in woodland strawberry [7]; this indicates that *gene06210* (*FveGRAS34*) participates in biological processes related to GAs in woodland strawberry. Lateral shoots of *Arabidopsis thaliana* and tillers of rice also develop from axillary buds; in *Arabidopsis thaliana* mutants of three *HAM* genes (*AtSCL6, AtSCL22*and *AtSCL27*) or one *LAS* gene (*AtLAS*), phenotypes with reduced lateral shoots could be observed during vegetative development [24,25,26,31], and mutation of the *LAS* subfamily gene *MOC1* in rice caused defective tiller formation [27,28,29]. Three *HAM* genes (*FveGRAS3, 35* and *52*) were found to be expressed in the crown, stolon and stolon tip (Figure 4A), indicating that these three genes may regulate stolon or branch crown development. However, the only one *LAS* gene, *FveGRAS32*, showed very low expression in all vegetative organs. A possible explanation for this result is that *FveGRAS32* does not participate in stolon or branch crown development, while an alternative possibility is that *FveGRAS32* was expressed in tissues or cells not tested in this study. In addition, we did not observe stolon-specific expression of *FveGRAS* genes, but many *FveGRAS* genes belonging to various subfamilies showed high expression in the crown, stolon, stolon tip, leaf and petiole (Figure 4A). Since photoperiod and temperature regulate axillary bud development into a stolon or branch crown, and as leaves are the main organ used to sense photoperiod and temperature, a signaling pathway from leaf to crown through the petiole may exist to regulate stolon or branch crown development [5,36,37]; thus, *FveGRAS* genes expressed in the crown, stolon, stolon tip, leaf or petiole may regulate stolon and branch crown initiation or elongation in woodland strawberry, although this requires further investigation.

### 3.3. Possible Roles of GRAS Genes in the Reproductive Organs of Woodland Strawberry

A large number of studies of *GRAS* genes have focused on their roles in the development of vegetative organs, but not much is known about the functions of GRAS protein during the reproductive stages from flower development to seed formation. *LlSCL* family genes were first functionally characterized in lily, where they were predominantly expressed during the premeiotic phase within the anther and specifically enhanced the activity of a meiosis-associated promoter during microsporogenesis [57]. However, we did not observe anther-specific expression of *LlSCL* genes in woodland strawberry; five *LlSCL* genes (*FveGRAS25, 23, 24, 19* and *21*) were highly expressed in both the carpel and anther (Figure 4B), suggesting that these genes may regulate carpel and anther development. All other subfamilies had some *GRAS* genes that were expressed in the carpel or anther or both (Figure 4B), indicating that many *FveGRAS* genes may participate in the development of those structures. Evidence supporting this finding is still lacking for *Arabidopsis thaliana* and rice, so further research is needed.

In addition, few studies have reported GRAS functions during seed development. For example, the expression of *AtSCR* and *AtSHR* in *Arabidopsis thaliana* can be detected at the heart stage of embryo development and may be responsible for meristem development during this stage [58,59]. In woodland strawberry, two *SCR* genes (F*veGRAS41* and *45*) and two *SHR* genes (*FveGRAS5* and *4*) showed expression in the embryo (Figure 5A), suggesting that these genes may have similar functions as *AtSCR* and *AtSHR* during embryo development. The *HAM* subfamily gene *AtSCL15* can be expressed in the seed coat, and its encoded protein can recruit HDA19 to prevent the transition from seed maturation to vegetative growth [60]; the orthologous gene *FveGRAS3* was not expressed in the embryo, but was expressed in ghost and the seed wall (Figure 5A), suggesting similar functions to *AtSCL15*. In rice, the *DLT* subfamily gene *GS6* negatively regulates grain size [32], while the *DLT* gene in woodland strawberry, *FveGRAS36*, was mainly expressed during the early stages of seed and receptacle (Figure 5A), revealing a role in early stage fruit development in woodland strawberry. Other genes in various *GRAS* subfamilies have not been studied in terms of their roles in seed development, including *HAM, SCL4/7, SCL3, PAT1* and *LlSCL* subfamily genes, but showed similar or obviously differing expression levels among the embryo, ghost and wall in woodland strawberry (Figure 5A); this suggests that many *FveGRAS* genes in various subfamilies may participate in seed development in woodland strawberry.

In contrast to the dry fruits of *Arabidopsis thaliana* and rice, woodland strawberry fruit is fleshy and develops from a receptacle with embedded seeds. ABA is the major phytohormone regulating strawberry fruit ripening [1], whereas tomato fruit ripening is regulated by ethylene [61]; thus, woodland strawberry is used as a model organism in studies of non-climacteric fruits. Therefore, we focused on the analysis of candidate *FveGRAS* genes, which may regulate woodland strawberry fruit ripening. At present, little is known about the GRAS function during fruit development and ripening, even in tomato (the model plant of climacteric fruits), although *GRAS* family genes have been identified in genome-wide analysis of tomato [48]. Knockdown of the *PAT1* subfamily gene *SlGRAS2* significantly reduced tomato fruit weight [62], silencing of D*ELLA* resulted in facultative parthenocarpy of tomato fruits [63], while overexpression of the *HAM* subfamily gene *SlGRAS24* and *SlGRAS24* resulted in reduced fruit set, smaller fruit size with fewer seeds [64,65], the homologs of these four genes in woodland strawberry *FveGRAS14, FvGRAS34* and *FveGRAS52* showed high expressions in all tested organs, including carpel, anther, seeds and immature fruits (Figure 5A). In addition, higher expression levels of tomato *GRAS* genes are generally seen in immature versus mature fruits [48], in accordance with results for woodland strawberry in the present study (Figure 5A); this suggests that most *GRAS* genes are involved in early fruit development. Five *SlGRAS* genes in tomato [48], and four *FveGRAS* genes (*FveGRAS3, 54, 12* and *46)* in woodland strawberry, showed dramatic increases in expression from the immature stage to the ripening stage (Figure 5B), indicating that these genes may play roles during the onset of ripening in tomato and woodland strawberry. These five tomato *GRAS* genes belong to the *HAM, PAT1, SCL3* and *SHR* subfamilies [48], and the four woodland strawberry *GRAS* genes belong to the *HAM* and *PAT1* subfamilies. In particular, the *SHR* family gene *SlGRAS38* and *SCL3* family gene *SlGRAS18* showed relatively strong and specific expression during fruit ripening, and have been reported as target genes of tomato-ripening key transcriptional regulator RIN [66,67]. In woodland strawberry, the expression of all *SHR* and *SCL3* family genes was negligible or decreased from the immature stage to the ripening stage (Figure 5B), indicating that the *SHR* and *SCL3*subfamily genes are likely not involved in woodland strawberry fruit ripening, which does not require ethylene. Furthermore, the *PAT1* family gene *FveGRAS54* showed more than a 10-fold increased expression, which is the largest increase during fruit ripening among *FveGRAS* genes (Figure 5B), indicating that *FveGRAS54* may play an important role in the fruit ripening of woodland strawberry; however, this requires validation. 

### 3.4. Response of GRAS Genes of Woodland Strawberry to Environmental Factors

Cultivated strawberry is a perennial rosette plant found throughout the Northern Hemisphere. During the long and warm days of summer, axillary buds in the crown of cultivated strawberry generally develop into stolons. When the days become shorter and temperatures decrease at the end of summer, stolon production ceases and axillary buds develop into branch crowns. During the short days of autumn, inflorescences are produced at the terminal apices of the main and branch crowns until growth and development cease in the winter. During spring, growth resumes and the inflorescences complete their development, followed by flowering and the development of fruits [36,37]. 

Light is a very important environmental factor for woodland strawberry growth and development; it not only regulates the differentiation of stolons, branch crowns and flowers, but also affects flowering and fruit growth, ripening and quality [36,37]. It has been demonstrated that members of the PAT1 subgroup of the GRAS family are downstream members of the phytochrome signal transduction pathway. AtPAT1 and AtSCL21 in *Arabidopsis thaliana* are closely related and can interact with each other; both are positive regulators of phytochrome A (phyA) signal transduction, because their mutants develop an elongated hypocotyl specifically under far-red light, which is a phyA-dependent trait [33,35]. Another PAT1 family protein, AtSCL13, is downstream of phytochrome B (phyB) and acts as a positive regulator of red-light signals. AtSCL13 can also modulate phyA signaling in a phyB-independent manner [34]. In this study, six PAT1 subfamily proteins were identified in woodland strawberry, which all had high expression levels in every organ and tissue tested (Figure 4 and Figure 5), suggesting that *PAT1* subfamily genes may participate in the regulation of light signals for many biological processes, including stolon and branch crown differentiation and fruit growth. More importantly, during fruit ripening, the expression levels of *FveGRAS46* and *FveGRAS12*, the most homologous genes of *AtPAT1*, *AtSCL21* and *AtSCL13*, increased; moreover, the expression of another *PAT1* subfamily gene, *FveGRAS54*, increased more than 10-fold (Figure 5B), indicating all three of these genes may play positive roles in light regulation on fruit ripening or the quality of woodland strawberry fruits.

Hot temperatures in summer and cold temperatures in winter can seriously affect cultivated strawberry vegetative and reproductive growth. To identify possible *FveGRAS* genes related to cold and heat tolerance, we assessed the responses of *FveGRAS* genes to heat and cold treatments. It has been reported that *GRAS* family genes can be regulated by abiotic stresses, including heat, salt and drought, and that overexpression of some *GRAS* genes can enhance tolerance to these stresses. For example, overexpression of the poplar *GRAS* gene *PeSCL7* enhanced salt and drought tolerance in *Arabidopsis thaliana* [68], overexpression of *BnLAS* from *Brassica napus* resulted in enhanced drought tolerance in *Arabidopsis thaliana* [69] and overexpression of *VaPAT1* from *Vitis amurensis* conferred cold, drought and high-salinity tolerance in *Arabidopsis thaliana* [70]. *FveGRAS2, FveGRAS32* and *FveGRAS14* in woodland strawberry are homologous genes of *PeSCL7*, *BnLAS* and *VaPAT1*, and we found that expression of *FveGRAS2* and *FveGRAS14* increased significantly with cold and heat treatments; in contrast, the change in *FveGRAS32* was negligible (Figure 6), suggesting that *FveGRAS2* and *FveGRAS14* may participate in cold and heat tolerance, while *FveGRAS32* does not. In addition, the *SCR* and *SHR* genes are involved in low-phosphate stress [71], *DELLA* genes are involved in many abiotic stresses including low temperature, phosphate starvation, osmotic stress, and high NO concentration [18,21], and the *LlSCL* family gene *OsGRAS23* is involved in drought stress [72]. However, the functions of other *GRAS* genes under abiotic stress conditions, especially heat and cold stresses, have not been studied. In our study, the expression levels of some *FveGRAS* genes showed opposite trends under heat and cold stresses (Figure 6), suggesting that these genes may regulate different pathways under heat and cold stresses. Meanwhile, some *FveGRAS* gene expression levels uniformly increased or decreased under both heat and cold stresses (Figure 6), suggesting that these genes may regulate the same response pathway to heat and cold stresses, and could possibly be used to enhance woodland strawberry tolerance to multiple environmental stresses. 

### 3.5. Response of GRAS Genes in Woodland Strawberry to GAs

As noted above, stolon propagation is the main reproductive method used during cultivated strawberry cultivation, and the stolon is an organ specific to cultivated strawberry not found in the model plants *Arabidopsis thaliana* and rice. Phytohormones GAs can regulate stolon initiation and elongation. Exogenous GA_3_ treatment promotes stolon formation, which is suppressed by GA biosynthetic inhibitor treatment [55,56]. The runnerless trait of the diploid woodland strawberry variety "Alpine" is due to mutation of the GA biosynthetic gene *GA20ox4*, which is expressed mainly in the axillary meristem dome and primordia, and in developing stolons [6]; this suggests that GA signaling genes, GA-regulated genes and regulators of GA biosynthesis may be involved in stolon initiation and elongation. GRAS proteins are important components of the GA signaling pathway, and the best studied GRAS proteins in GA signaling are DELLAs, which are master repressors that can directly interact with GA receptors to inhibit GA signaling [18]. In woodland strawberry, one GRAS protein with a full DELLA motif can regulate the development of internodes, flowering shoots, leaves and stolons [6], and expression of this gene (*FveGRAS34*) was induced by GA_3_ treatment in this study. Some other *GRAS* genes have also been functionally characterized in terms of their roles in GA signaling in *Arabidopsis thaliana* and rice, but not in woodland strawberry. For example, AtSCL3 promotes GA signaling by antagonizing the master growth repressor DELLA during seed germination and seedling growth in *Arabidopsis thaliana*, while *AtSCL3* expression is induced by DELLAs and repressed by GA [73]. As an orthologous gene of *AtSCL3*, *FveGRAS49* expression was also repressed by GA_3_ treatment in this study (Figure 8), suggesting conserved function of *FveGRAS49* with *AtSCL3* in GA signal transduction. In addition, overexpression of the *SCL4/7* subfamily gene *Ha-GRASL* from sunflower reduced the metabolic flow of GAs and increased axillary meristem outgrowth in *Arabidopsis thaliana* [74], while the *SCL4/7* subfamily gene *FveGRAS2* in woodland strawberry was induced by GA_3_ treatment (Figure 8). In addition to GAs, *GRAS* family genes are also involved in other hormones. DELLA proteins also mediate auxin, BR, JA and ethylene signaling, thereby acting as a major hub in hormonal signaling [21]. Overexpression of the tomato *HAM* subfamily gene *SlGRAS24* and *SlGRAS40*, and *PAT1* subfamily gene *SlGRAS7* all affected multiple agronomic traits through regulation of GA and auxin signaling, and the expression of *SlGRAS40* and *SlGRAS7* were down-regulated, but *SlGRAS24* was up-regulated by auxin and GA treatments [64,65,75], while in woodland strawberry, *SlGRAS7* homologous gene F*veGRAS46,* and *SlGRAS24* and *SlGRAS40* homologous gene *FveGRAS52* were all down-regulated after GA_3_ treatment (Figure 8). The rice *DLT* subfamily gene *GS6* not only regulates GA biosynthesis, but also plays a role in BR signaling [76], and the rice *SCL4/7* subfamily gene O*sGRAS19* (*OsGRAS18* in this study) acts as a positive regulator in BR signaling [77], suggesting that *GRAS* genes are involved in crosstalk among hormonal signaling pathways. In this study, the expression levels of 26 *GRAS* genes in woodland strawberry either increased or decreased after GA_3_ treatment (Figure 8), indicating that these genes may play roles in biological processes related to GA biosynthesis, metabolism or signaling, such as stolon initiation and elongation. In particular, the expression of *FveGRAS14* showed the largest increase, of more than 10-fold, after GA_3_ treatment (Figure 8), This result, together with the *FveGRAS14* orthologous gene *AtPAT1* in *Arabidopsis thaliana* being a positive regulator of phyA signal transduction, and the photoperiod regulating stolon formation in woodland strawberry, led us to speculate that *FveGRAS14* may play a role in the regulation of stolon formation by photoperiod. However, the effects of *FveGRAS* genes on GAs and other hormones, and their roles in the growth and development of woodland strawberry, require further investigation.

## 4. Materials and Methods 

### 4.1. Identification and Phylogenetic Analysis of GRAS Proteins

The protein databases and annotation information of *Arabidopsis thaliana* and rice (*Oryza sativa*) were downloaded from Phytozome (https://phytozome.jgi.doe.gov/pz/portal.html. Access date: 16 May 2019), and the protein database and corresponding annotation of woodland strawberry (*F.vesca*) was downloaded from GDR (https://www.rosaceae.org/species/fragaria/fragaria_vesca. Access date: 16 May 2019). The full-alignment sequences of the GRAS domain (PF03514.13) was downloaded from the Pfam database, HMMER software was used to identify similar proteins in *Arabidopsis thaliana*, rice and woodland strawberry with an E-value cut-off of 1 × e^−4^ using PF03514.13 as query. The longest proteins were selected when there was alternative splicing, and the GRAS domain of identified proteins was confirmed using the Pfam (http://pfam.xfam.org/search. Access date: 3 June 2019) and SMART (http://smart.embl-heidelberg.de/ Access date: 3 June 2019). The 54 putative *FveGRAS* genes were renamed as *FveGRAS1* to *FveGRAS54* according to their chromosomal locations. 

34 and 60 GRAS proteins were identified from *Arabidopsis thaliana* and rice respectively, but one *Arabidopsis thaliana* protein and ten rice proteins containing partial GRAS domains were considered as pseudogenes. Therefore, the protein sequences of other 33 and 50 GRAS proteins from *Arabidopsis thaliana* and rice, together with 54 GRAS proteins from woodland strawberry were aligned using the ClustalX2.0 program with the default settings. A phylogenetic tree based on the alignment was constructed using MEGA6.0 by the NJ (neighbor-joining) method with the bootstrap test replicated 1000 times.

### 4.2. Conserved Domain and Motif Analysis of GRAS Proteins

All complete protein sequences of GRASs were used to analyze conserved domains and motifs. The conserved domains were identified by Pfam website, and the conserved motifs were identified by MEME website (Version 5.0.5, http://meme-suite.org/tools/meme. Access date: 4 June 2019) [78], with the maximum number of motifs was 30, and other parameters were default settings. The illustration containing conserved domains and motifs was constructed using TBtools software (Version No.0.66763, South China Agricultural University, Guangzhou, China) [79].

### 4.3. Identification of Orthologs, Coorthologs and Paralogs of GRAS Genes

Orthologous, coorthologous and paralogous gene pairs were identified by submitting all complete protein sequences of GRAS from *Arabidopsis thaliana*, rice and woodland strawberry to OrthoMCL software (Version 2.0, University of Pennsylvania, Philadelphia, USA) [80]. Illustrations of orthologous, coorthologous and paralogous gene pairs among the three species were constructed using Circos software (Canada’s Michael Smith Genome Sciences Center, Vancouver, Canada) [81].

### 4.4. Plant Materials and Cold, Heat and GA_3_Treatments

The genome-sequenced diploid woodland strawberry “Hawaii 4” (*F.vesca*) was used as plant material. “Hawaii 4” seeds were sterilized and sowed as previously studied [82], and the seedlings were grown in a climate chamber under a 16 h light (22 °C) with 15,000 Lux irradiance and 8 h of dark (20 °C) for about two months. Then the seedlings were transferred into pots containing a mixture of perlite, vermiculite and sphagnum (ratio, 1:2:3) in January 2019, and grown in a glass-enclosed greenhouse of Nanjing Agricultural University under native photoperiod. On 23th April 2019, we collected immature fruits with seeds about 8–15 DPA, stolon tips and fully open flowersfrom about 100 plants because of limited organs. Then the root was sufficiently washed by running water to remove soils, the clean roots, crowns, leaves, petioles and stolons were sampled from 24 plants. All of the samples were rapidly frozen in liquid nitrogen, and then stored at −80 °C for RNA extraction. Roots, crowns, leaves, petioles, stolons and immature fruits had three biological replicates, and stolon tips and fully open flowers had two biological replicates because of limited samples.

For cold, heat and GA_3_ treatments, “Hawaii 4” seeds were sterilized and sowed as previously studied [82], and the seedlings were grown in a climate chamber under 16 h of light (22 °C) with 15000 Lux irradiance and 8 h of dark (20 °C) for about two months. To avoid a photoperiod effect on gene expression during treatments, the growth conditions were reset as 24 h light (22 °C), and the seedlings were re-adapted for three days before treatments. Afterwards, seedlings were transferred to another chamber maintained at 40 °C for heat treatment and at 4 °C for cold treatment. For the GA_3_ treatment, the seedlings were sprayed with 50mg/L GA_3_ solution [5,6]. At 0, 2, 6, 12, 24 and 48 h after treatments, the whole seedlings with roots were collected, rapidly frozen in liquid nitrogen, and then stored at −80 °C for RNA extraction. Three biological replicates were performed.

### 4.5. RNA Extraction, Transcriptome Sequencing and Data Analysis

RNA extraction and transcriptome sequencing were performed according to Gu et al. (2019) [38]. Afterward, Raw data from transcriptome sequencing were firstly processed through in-house perl scripts. Clean data were obtained by removing reads with adapter, ploy-N and low quality from raw data. Q20, Q30 and GC content of the clean data were calculated at the same time. Reference genome of woodland strawberry *F.vesca* v4.0.a1 and gene annotation files *F.vesca* v4.0.a2 were downloaded from website (https://www.rosaceae.org/species/fragaria/fragaria_vesca. Access date: 16 May 2019). The index of the reference genome was built by Hisat2 v2.0.4 and paired-end clean reads were aligned to the reference genome by Hisat2 v2.0.4. The reads numbers mapped to each gene was counted by HTSeq v0.9.1, then FPKM value of each gene was calculated based on the length of the gene and reads count mapped to this gene.

### 4.6. Heat Map Construction of FveGRAS Gene Expressions 

The reads per kilobase per million (RPKM) values of transcriptome data in Figure 4B and Figure 5 of woodland strawberry were downloaded from Li et al. (2019) [43]. The FPKM values of *FveGRAS* genes in Figure 4A, Figure 6 and Figure 7 were selected from our own transcriptome data. Then RPKM and FPKM values were transformed in log2 level, and heat maps were constructed by MeV4.8 software.

## 5. Conclusions

In this study, we identified 54 FveGRAS proteins in woodland strawberry, and divided them into 14 subfamilies. Phylogenetic analysis, motif composition, orthologous and paralogous analysis were performed to study the genetic relationship among woodland strawberry, *Arabidopsis thaliana* and rice. The RNA-seq analysis showed that *FveGRAS* genes were expressed with different degrees in different organs and tissues. Sixteen genes showed decreased expression, while four genes showed increased expression during fruit ripening. In addition, around half of the *FveGRAS* genes displayed increased or decreased expression to some extent under cold, heat and GA_3_ treatments. A few *FveGRAS* genes were predicted as candidate genes to study their functions in stolon formation, fruit ripening and abiotic stresses.

## Supplementary Materials

Supplementary materials can be found at https://www.mdpi.com/1422-0067/20/18/4593/s1. Supplementary Table S1: Identification and characterization of GRAS proteins in woodland strawberry; Supplementary Table S2: Motif composition of GRAS family in woodland strawberry; Supplementary Table S3: Orthologs and co-orthologs of GRAS proteins among woodland strawberry, Arabidopsis and rice; Supplementary Table S4: Paralogs of GRAS proteins in woodland strawberry; Supplementary Table S5: The FPKM value of GRAS genes in different organs of woodland strawberry; Supplementary Table S6: The RPKM value of GRAS genes in the flowers of woodland strawberry; Supplementary Table S7: The RPKM value of GRAS genes in the flowers, early-stage fruits and ripening fruits of woodland strawberry; Supplementary Table S8: The RPKM value of GRAS genes in the ripening fruits of woodland strawberry; Supplementary Table S9: The FPKM value of GRAS genes uder cold and heat treatments of woodland strawberry seedlings; Supplementary Table S10: The FPKM value of GRAS genes uder GA_3_ treatment of woodland strawberry seedlings.

## Abbreviations

CO	CONSTANS
SOC1	SUPPRESSOR OF OVEREXPRESSION OF CONSTANS1
GA20ox4	Gibberellin 20-oxidase 4
ABA	Abscisic Acid
GAI	GIBBERELLIN-INSENSITIVE
RGA	REPRESSOR OF GA1-3
SCR	SCARECROW
LHR	Leucine Heptad Repeat
GAs	Gibberellins
BRs	Brassinosteroids
CKs	Cytokinins
JA	Jasmonate
SLs	Strigolactones
Chr	Chromosome
FPKM	Fragments Per Kilobase Per Million
HGT	Horizontal Gene Transfer
PhyA	Phytochrome A
PhyB	Phytochrome B
